# Myeloid Sarcoma: Clinicopathologic, Cytogenetic, and Outcome Analysis of 21 Adult Patients

**DOI:** 10.4061/2011/523168

**Published:** 2010-12-26

**Authors:** Hani Al-Khateeb, Ahmed Badheeb, Husam Haddad, Lina Marei, Salah Abbasi

**Affiliations:** ^1^Medical Oncology Department, King Hussein Cancer Center, Al-Jubeiha, Amman 11941, Jordan; ^2^Pathology Department, King Hussein Cancer Center, Al-Jubeiha, Amman 11941, Jordan

## Abstract

Myeloid sarcoma (MS) is a neoplasm of immature granulocytes, monocytes, or both involving any extramedullary site. Twenty one patients with MS at diagnosis who were treated at King Hussein Cancer Center in Jordan were included in this retrospective study with a male to female ratio of 2 : 1. The most common site was the reticuloendothelial system. The most common morphology subtype was M2 (38%) and the most frequent chromosomal abnormality was trisomy 8. Twenty patients received induction chemotherapy; only 14 (70%) achieved complete remission. Median survival time was 24.7 months for the whole group and 58.6 months for patients who underwent allogenic bone marrow transplant. This paper showed that MS has frequent M2 morphology, carries chromosomal aberrations other than t(8;21), and requires aggressive therapy as a front line approach.

## 1. Introduction

Myeloid sarcoma (MS) is a tumorous aggregate of malignant immature granulocytes, monocytes, or both involving any extramedullary site. Although first described by Burns [1] in 1811, it was King [2] in 1853 who coined the term chloroma based on the green color of the tumorous mass attributable to the enzyme myeloperoxidase. MS may occur de novo in the absence of any past history or current bone marrow involvement by acute myeloid leukemia (AML), myelodysplastic syndrome (MDS), or myeloproliferative disorder (MPD) [3]. This primary form of MS is relatively rare. On the other hand, secondary MS (defined as the occurrence of MS manifestation in patients with previous or current bone marrow involvement by AML, MDS, or MPD) occurs in approximately 1.4% to 9% of patients with AML [4, 5]. MS is frequently mistaken for non-Hodgkin's lymphoma, small round cell tumors (neuroblastoma, rhabdomyosarcoma, Ewing sarcoma, and medulloblastoma), and undifferentiated carcinoma, which may cause misdiagnosis in about 50% of cases when immunohistochemistry is not used [6].

The present study was designated to evaluate the lineage differentiation of neoplastic cells in MS by immunohistochemistry and to correlate the results with the clinicopathological features, cytogenetics, and treatment outcomes.

## 2. Patients and Methods

Twenty one adult (18 years of age or more) patients with a histologic diagnosis of MS at presentation, who were managed at KHCC in Jordan between 2004 and 2008, were included in the present study. The study was approved by the institutional review board at KHCC.

The charts of these patients were reviewed retrospectively for data collection including age, sex, anatomic site of involvement, French-American-British subtype, immunohistochemical evaluation, bone marrow biopsy results, history of AML, MDS, or MPD, chromosomal studies, type of chemotherapy used for induction, response to induction therapy, and median survival time.

The initial diagnosis of MS was made on core biopsy and/or surgical specimens. Immunohistochemical studies were performed at KHCC laboratory. Chromosomal analysis was performed on tumor specimens (from the bone marrow in patients with synchronous bone marrow infiltration and from the extramedullary site of involvement in de novo MS cases) of 20 patients at diagnosis. Cells were cultured in RPMI 1640 medium with 20% fetal bovine serum for 24 hours and 48 hours, respectively. Metaphase cells were analyzed following standard G-banding method. Karyotypes were interpreted according to the international system for human cytogenetic nomenclature.

### 2.1. Statistical Analysis

Median survival time was calculated from the date of diagnosis of MS until death or date of the last contact for living patients. The univariate association between individual clinical features and median survival was determined with the log-rank or Wilcoxon's test, whichever appropriate. Factors independently associated with median survival were identified in multivariate analysis by the Cox proportional hazards regression model.

## 3. Results

Demographic, clinicopathologic, and cytogenetic findings are shown in Table 1.

### 3.1. Demographic Data

The study included 14 male and 7 female patients with an age range of 21 to 77 years (mean, 44.4 years) and a male to female ratio of 2 : 1. De novo MS occurred in 8 (38.1%) cases. Thirteen patients with secondary MS manifestation had synchronous bone marrow infiltration by AML or MPD at time of presentation; one case had chronic myelogenous leukemia (blast phase), 6 cases had AML/M4, 3 cases had AML/M2, 2 cases had AML/M5, and one case had AML/M0.

### 3.2. Sites of Involvement

The most common extramedullary sites of involvement were the reticuloendothelial system (7 cases), followed by the thoracic cavity (4 cases) and the skin (4 cases).

### 3.3. Pathology Findings

The most commonly expressed immunohistochemical marker was CD34, which was present in all cases of MS (100%). CD117 was positive in 19 (90%) cases, and myeloperoxidase was variably positive in 18 (85%) cases. Other positive markers were, CD68 (KP-1) in 18 (85%) cases, CD68 (PG-M1) in 9 (43%) cases, CD15 in 17 (81%) cases, lysozyme in 8 (38%) cases, CD45 in 7 (33%) cases, CD43 in 2 (10%) cases, and TdT in 1 (5%) case.

The most common French-American-British subtypes were M2 (8 cases, 38%) and M4 (7 cases, 33%). The rest were M0, M1, and M5, two cases each.

The histology of all 8 de novo cases of MS according to the most recent WHO criteria [7] was AML-not otherwise specified (4 cases had AML with maturation, 2 cases had AML without maturation, one case had acute myelomonocytic leukemia, and one case had AML with minimal differentiation).

All MS cases were morphologically and immunophenotypically analogous to their leukemia counterparts.

### 3.4. Karyotypes

Chromosomal studies were done for 20 patients only, and abnormalities were observed in 11 (55%) cases examined. Nine cases had more than one chromosomal aberration. The most common aberration was the presence of an extra chromosome 8 (20%). Translocation (8;21) was demonstrated in one case only. Five of the eight de novo EML cases had chromosomal aberrations (all had more than one aberration). Molecular data for the FLT3 mutation was available for 16 patients only and was negative in all cases.

### 3.5. Therapy Results

Twenty patients were treated with induction chemotherapy (7  +  3 in 18 cases, and high dose cytarabine in 2 cases). One patient received best supportive care only because of poor performance status. The median follow-up time for the whole patient's population was 56 months. Fourteen (70%) patients achieved complete remission (CR1) after first induction chemotherapy. Four patients in CR1 received myeloablative allogenic bone marrow transplant (AlloBMT) as consolidation therapy, and two patients received AlloBMT after achieving second complete remission (CR2) after relapse. All patients who received AlloBMT had conditioning regimen including total body irradiation and cyclophosphamide, and all patients received HLA-matched related donors (HLA-identical siblings). One patient who was transplanted in CR1 developed acute graft versus host disease (GVHD), and two patients (one transplanted in CR1 and one transplanted in CR2) developed chronic GVHD.

The median survival time was 24.7 months (range, 1.5 to 86) for the whole group, 34 months for patients who achieved CR1, 28 months for de novo MS cases, 22.7 months for secondary MS cases, and for patients who underwent AlloBMT (four patients received AlloBMT as initial therapy, and two as salvage therapy), their median survival time was 58.6 months.

Response to therapy and median survival were not influenced by any of the following factors: age, sex, anatomic site of involvement, histotype, phenotype, cytogenetics, and presence of underlying AML, or MPD, but median survival time was significantly higher for patients who underwent myeloablative AlloBMT (*P* = .0001).

## 4. Discussion

MS is a rare extramedullary tumorous aggregate of malignant myeloid precursor cells [7]. It usually occurs in patients with AML, MDS, or MPD, but it may occur de novo or rarely precede peripheral blood or bone marrow involvement, presenting a diagnostic challenge. In our study, all patients were adults with a male predominance. Similar to previous report [8], the mean age at diagnosis was 44.4 years, and the neoplasm occurred in different locations. Although the reticuloendothelial system was the most common site of extramedullary involvement, it was unexpectedly more frequent in the thoracic cavity than previously reported.

The correct diagnosis of MS is important for adequate therapy, which is often delayed because of a high misdiagnosis rate. The differential diagnosis of MS includes several entities, the majority of which can be readily distinguished using a combination of morphologic and immunohistochemical evaluation. An incomplete workup may be misleading because B-cell or T-cell lymphoma and MS share morphologic similarities and both express some leukocyte antigens, such as CD43, and CD45. CD34 is the human hematopoietic progenitor antigen and is, expressed in nearly 40% of de novo acute leukemia cases. Our study showed 100% expression of CD34 by all MS cases, which makes it the most sensitive marker in MS. In contrast to previous studies which showed 100% expression of CD43 in MS [9], our results showed only two cases with CD43 expression.

Similar to other studies [10], we showed that MS, in adults, is most frequently associated with French-American-British subtypes M2 and M4, which is probably one of the most important risk factors for extramedullary involvement in patients with AML.

MS has been described in association with a variety of chromosomal aberrations, in particular, t(8;21), inv(16), and +8 [3, 11]. Our study showed a high incidence of chromosomal aberrations among our MS population (55%), with most of the cases showing more than one abnormality. We demonstrated a high frequency of chromosomal aberrations in de novo MS cases as well (5/8, 62.5%). In agreement with previous reports [11], chromosome 8 abnormalities, especially an extra chromosome 8, was the most common genetic aberration among our population of MS patients.

Other studies have indicated that the behavior of MS is dramatic irrespective of presentation, age, sex, phenotype, and cytogenetics [12]. This is demonstrated in our results which showed a short median survival time of 24.7 months only. We found no significant difference in median survival or CR1 rate between patients with de novo disease and those with tumors related to AML or MPD.

Although not based on a randomized clinical trial, our study provides a strong support to the concept that patients with MS should undergo aggressive therapy, including AlloBMT. Patients who received myeloablative AlloBMT achieved a significantly higher median survival time than patients who did not undergo bone marrow transplant (58.6 months versus 11.1 months, resp.; *P* = .0001).

Future studies should evaluate the prognostic significance of MS in randomized clinical trials to test the need for allogenic bone marrow transplant in these patients.

## 5. Conclusion

MS among Jordanian acute leukemia patients has frequent M2 morphology, carries chromosomal aberrations other than t(8;21), and requires aggressive therapy as front line approach.

##  Conflict of Interests

The authors declare that they have no conflict of interests.

## Figures and Tables

**Table 1:** Characteristics of 21 adult patients with myeloid sarcoma.

No.	Age	Sex	Site	FAB	BMBx	Karyotype
1	43	M	LN/spleen	M2	CML/BP	49, xy, t(9;22), t(12;18), +8, +19, +20
2	37	F	Pleura	M4	AML/M4	46, xx
3	37	F	Pleura	M0	AML/M0	46, xx/47, xy, t(1;3), del5, +8, +3, +4, −12
4	45	M	LN	M0	normal	46, xx/45, xy, t(7;11), t(12;18)
5	23	M	CNS	M2	AML/M2	46, xy, t(9;22)
6	64	M	CNS	M4	AML/M4	46, xy
7	28	F	Vulva	M5	AML/M5	46, xx, t(3;11)(p21;q23)
8	21	F	Lumbar spine-extradural	M4	AML/M4	46, xx, t(8;21), +8, −7
9	72	M	LN	M4	AML/M4	46, xy
10	76	F	LN	M2	normal	Not available
11	43	M	Mediastin-al LN	M5	AML/M5	46, xy, i(1), t(1;1), −2, −5, −6, +8, −11, −12, −15, −17, −19, −22
12	54	M	Skin	M2	AML/M2	46, xy
13	35	M	Head of femur	M4	AML/M4	46, xy
14	77	M	Orbit	M2	normal	46, xy/47, xy, t(1;3), +15
15	63	M	Mediastin-al LN	M2	normal	46, xy/49, xy/50, xy, +2
16	43	M	Pleura	M2	normal	46, xy
17	39	M	Perianal skin	M4	AML/M4	46, xy
18	34	M	CNS	M1	normal	45, xy, t(9;22), −7 *
19	73	M	LN/spleen	M1	normal	47, xy, del5, −3, −7, +22
20	69	F	Pleura	M2	AML/M2	46, xx
21	31	F	Skin mass (groin)	M4	normal	46, xx

Abbreviations: FAB: French-American-British classification; BMBx: bone marrow biopsy; M: male; F: female; LN: lymph node; CNS: central nervous system; CML/BP: chronic myeloid leukemia/blastic phase; AML: acute myeloid leukemia.

*Real-time quantitative polymerase chain reaction (RQ-PCR) test for t(9;22) in the bone marrow was negative.
